# Efficacy of Cladribine Tablets as a Treatment for People With Multiple Sclerosis: Protocol for the CLOBAS Study (Cladribine, a Multicenter, Long-term Efficacy and Biomarker Australian Study)

**DOI:** 10.2196/24969

**Published:** 2021-10-19

**Authors:** Vicki E Maltby, Rodney A Lea, Mastura Monif, Marzena J Fabis-Pedrini, Katherine Buzzard, Tomas Kalincik, Allan G Kermode, Bruce Taylor, Suzanne Hodgkinson, Pamela McCombe, Helmut Butzkueven, Michael Barnett, Jeannette Lechner-Scott

**Affiliations:** 1 Department of Neurology John Hunter Hospital New Lambton Heights Australia; 2 School for Medicine and Public Health University of Newcastle Callaghan Australia; 3 Hunter Medical Research Institute New Lambton Heights Australia; 4 Institute of Health and Biomedical Innovations Genomics Research Centre Queensland University of Technology Kelvin Grove Australia; 5 Department of Neurosciences, Eastern Health Clinical School Monash University Box Hill Hospital Melbourne Australia; 6 Department of Neurology Alfred Health Melbourne Australia; 7 Department of Neurology Melbourne Multiple Sclerosis Centre Melbourne Health Melbourne Australia; 8 Centre for Neuromuscular and Neurological Disorders Perron Institute for Neurological and Translational Science University of Western Australia Perth Australia; 9 Clinical Outcomes Research (CORe) Unit Department of Medicine University of Melbourne Melbourne Australia; 10 Institute for Immunology and Infectious Disease Murdoch University Perth Australia; 11 Menzies Institute for Medical Research University of Tasmania Hobart Australia; 12 Department of Medicine University of New South Wales Sydney Australia; 13 Department of Neurology Liverpool Hospital Sydney Australia; 14 Immune Tolerance Laboratory Ingham Institute Sydney Australia; 15 Centre for Clinical Research University of Queensland Brisbane Australia; 16 Brain and Mind Centre University of Sydney Sydney Australia; 17 Sydney Neuroimaging Analysis Centre Sydney Australia

**Keywords:** multiple sclerosis, cladribine, biomarkers

## Abstract

**Background:**

Cladribine tablets (marketed as Mavenclad) are a new oral therapy, which has recently been listed on the pharmaceutical benefits scheme in Australia for the treatment of relapsing multiple sclerosis (MS). The current dosing schedule is for 2 courses given a year apart, which has been shown to be effective for treatment of MS for up to 4 years in 75% of patients (based on annualized relapse rate). However, the reinitiation of therapy after year 4 has not been studied.

**Objective:**

This study aims to evaluate the safety and efficacy of cladribine tablets over a 6-year period, according to no evidence of disease activity 3.

**Methods:**

This will be a multicenter, 6-year, phase IV, low interventional, observational study that incorporates clinical, hematological, biochemical, epigenetic, radiological and cognitive biomarkers of disease. Participants considered for treatment with cladribine as part of their routine clinical care will be consented to take part in the study. They will be monitored at regular intervals during the initial course of medication administration in years 1 and 2. After year 3, patients will have the option of redosing, if clinically indicated, or to switch to another disease-modifying therapy. Throughout the duration of the study, we will assess blood-based biomarkers including lymphocyte subsets, serum neurofilament light chain, DNA methylation, and RNA analysis as well as magnetic resonance imaging findings (brain volume and/or lesion load) and cognitive performance.

**Results:**

This study has been approved by the Hunter New England Local Health District Human Research Ethics Committee. Recruitment began in March of 2019 and was completed by June 2021.

**Conclusions:**

This will be the first long-term efficacy trial of cladribine, which offers reinitiation of therapy in the 3rd year, based on disease activity, after the initial 2 courses. We expect that this study will indicate whether any of the assessed biomarkers can be used to predict treatment efficacy or the need for future reinitiation of cladribine in people with MS.

**Trial Registration:**

This study is registered with the Australian and New Zealand Clinical Trials Registry (ACTRN12619000257167) with Universal Trial Number (U1111-1228-2165).

**International Registered Report Identifier (IRRID):**

DERR1-10.2196/24969

## Introduction

### Overview

Multiple sclerosis (MS) is a common, immune-mediated, demyelinating disease that affects the central nervous system. MS has a highly variable course; therefore, patient-specific treatment decisions are becoming increasingly important. Currently, we have no way to differentiate between the patients who will acquire rapid disability progression and the ones who will remain stable over several years. Most studies point toward early intervention giving a better long-term outcome; however, long-term immunosuppression is associated with increased adverse risk [1,2]. Generally, MS therapies are long-term and some have a demonstrated rebound phenomenon precluding them from being stopped for long periods of time, such as fingolimod [3] and natalizumab [4]. However, there are currently 2 drugs that can potentially be used for immune reconstitution treatment: alemtuzumab and cladribine.

Alemtuzumab is administered in 2 courses, via infusion, 1 year apart. In the Comparison of Alemtuzumab and Rebif Efficacy in Multiple Sclerosis Study (CARE-MS) extension trial, a redosing scheme was introduced, with up to 4 additional doses being offered in the event of recurring clinical activity [5]. Cladribine is a synthetic purine analog, and its mechanism of action is thought to be primarily via induction of apoptosis in lymphocytes [6]. It was shown in a phase III trial (A Safety and Efficacy Study of Oral Cladribine in Subjects with Relapsing-Remitting Multiple Sclerosis; CLARITY) to be highly effective in controlling disease activity [7-10]. With a no evidence of disease activity 3 (NEDA 3) rate of 44% over 2 years [7], it is placed in the range of the most efficacious disease-modifying therapies (DMTs) along with alemtuzumab (39%) [11,12] and ocrelizumab (47%) [13]. Furthermore, after 2 years of treatment and 2 years of follow-up (treatment free), 75% of patients with MS remained relapse free [8,14]. However, clinical stability beyond year 4, and additional doses of cladribine tablets based on clinical activity, has not yet been studied.

In the study discussed below*,* we will offer additional doses of cladribine tablets after 3 years. We aim to compare the clinical outcomes of patients with MS who received additional doses of cladribine with those who changed DMT. We also aim to identify biomarkers for disease control that can be used for treatment decisions, such as redosing with cladribine tablets versus change of DMT.

### Justification of Outcome Measures: NEDA

With the introduction of an ever-increasing number of DMTs, our treatment goals have shifted from simply reducing relapses to achieving *NEDA*.

In MS, NEDA 3 is defined as no clinically confirmed progression, no relapse, and no new or enlarging or gadolinium (Gd)-enhancing lesions [15]. This has since been expanded to NEDA 4, which includes brain atrophy [16]. Although our current treatments are increasingly effective, only 30% of patients with MS reach NEDA 4 after 2 years [17]. The rate of NEDA 4 in patients with MS treated with cladribine has not yet been established.

### Magnetic Resonance Imaging for Assessing Progression of MS

Magnetic resonance imaging (MRI) has been established as the most reliable indicator of long-term outcomes. Sormani et al [18] analyzed data from 13 large clinical trials, including 13,500 patients with MS, to show that new T2 lesions can predict disability progression. This prediction was significantly improved when combined with brain atrophy [18]. Some of the high efficacy treatments can change the rate of brain volume loss to that seen in the normal aging population [19]. As MRI technology advances, it has become clear that some substructures such as thalamic volume, lateral ventricle, and gray or white matter volume might be even more sensitive and correlate better with clinical outcomes [20].

### Lymphocytes as Biomarkers

Blood-based biomarkers are attractive because of their ease of collection and cost-effectiveness. In addition, they may better reflect or even predict disease activity. Lymphocyte subsets may be good markers of disease stability, as many currently used therapies act by restoring the balance of immune cells in the periphery to a *healthy* state. Therapies affecting the B-cell population have been described as effective treatments for MS [21]. This may be related to the suppression of memory B cells. This theory is supported by the failure of Atacicept and tumor necrosis factor α inhibitors in treating MS, both of which result in disease activation [21]. In both ORACLE-MS and CLARITY, cladribine has been shown to markedly reduce B cells, while having a more modest reduction in T cells and natural killer cells [22,23]. A more recent study of the CLARITY cohort further characterized this reduction and demonstrated that memory B cells were the predominantly affected subtype [24].

These studies investigated lymphocyte subsets over the course of 1-2 years. Therefore, a long-term investigation of lymphocyte subsets in response to cladribine treatment is warranted.

### Cognitive Dysfunction in MS

Cognitive impairment is a prevalent symptom in MS, with rates of approximately 40%-70% [25]. The severity of cognitive deficits varies, but unlike the physical symptoms associated with MS, cognitive deficits are unlikely to remit and are associated with a higher risk of progression [26-28]. A recent systematic review evaluated the literature on cognitive impairment and employment status and found a consensus that patients with MS who are unemployed or have reduced work hours record weaker cognitive scores [29]. Loss of employment is a major concern for patients with MS, particularly as the disease is often diagnosed in people of working age, who are just establishing careers and families. Despite this, cognitive testing is often disregarded in terms of clinical trial outcomes. Several tools have been developed to evaluate cognitive function, including the Brief Cognitive Assessment for Multiple Sclerosis (BICAMS) [30]. The BICAMS is quick and easy to administer and has recently been validated in the Australian population [31].

### Global Epigenetic Profiles in MS

Epigenetics is a rapidly developing area of medical research and refers to the potentially reversible regulation of genomic functions, particularly gene expression. This provides a mechanism whereby an organism can dynamically respond to a change in its environment (eg, DMTs) and alter its gene expression accordingly. DNA methylation is a well-characterized, relatively easy to study epigenetic modification and generally refers to the addition of a methyl group to a cytosine base, followed by a guanidine (referred to as a CpG dinucleotide).

We, and others, have described differential DNA methylation profiles between healthy controls and patients with MS, as well as between relapsing remitting MS and secondary progressive MS [32-38]. In addition, we and others have performed longitudinal studies that found epigenetic profiles are altered after dimethyl fumarate treatment [39-41], making it a potentially specific biomarker for treatment response.

### Neurofilaments are a Promising Biomarker

Neurofilament (NfL) light chains are found in neuronal cells but are shed into the cerebrospinal fluid upon neuronal damage and are detectable in the peripheral blood. Increased serum NfL levels have been identified in patients with MS compared with that in healthy controls [42]. These levels show a strong correlation not only with cerebrospinal fluid NfL levels but also with the presence and activity of focal lesions and clinical outcomes [42]. This makes them a promising biomarker not only of disease activity but also disease progression. These may be useful indicators of the need for additional courses of treatment.

### Objective

The primary objective of the study is to evaluate the safety and efficacy of cladribine tablets over a 6-year period according to NEDA 3. We will also evaluate clinical outcomes and cognition over a 6-year period, blood-based and MRI-based biomarkers for their ability to predict treatment response, disease activity, and the need to redose cladribine subsequent to the 2-year, 2 initial courses.

## Methods

### Study Design

This study is a multicenter, 6-year, phase IV, low interventional trial. Enrollment of 150 patients with MS was planned across 9 specialist MS clinics in Australia. The Therapeutic Goods Australia approved a cumulative dose of cladribine (10 mg) tablets as 3.5 mg/kg body weight over 2 years administered as 1 treatment course of 1.75 mg/kg per year. After 3 years, patients with MS in consultation with their health care provider will discuss the option of redosing with cladribine tablets, if clinically indicated (by relapse or new MRI activity), switch to another DMT, or continue without change (without commencement of any DMT). For those who will be continued on cladribine, additional courses will be administered as per the previous dosing of 1.75 mg/kg per year.

### Eligibility Criteria

Patients must meet the criteria as described below (Textbox 1).

**Inclusion and exclusion criteria.**

**Inclusion criteria**

- Participants must be eligible for and already intend to commence cladribine tablets in accordance with the Australian Product Information (PI). Cladribine tablets are indicated for relapsing remitting patients with multiple sclerosis who do not have HIV infection, active chronic infection, are immunocompromised, have severe renal impairment, or are pregnant or breastfeeding.
- Participants must have the ability to understand the purpose and risks of the study, as outlined in the patient informed consent form and provide signed and informed consent and authorization to use protected health information in accordance with national and local privacy regulations.
- The participants must meet the McDonald criteria [43] for the diagnosis of relapsing remitting multiple sclerosis.
- Male or female participants aged 18-70 years.
- Be able to provide details for or consent to provide access to a stored minimum data set (ie, demographics, date of diagnosis, relapse information, and baseline expanded disability status scale score).
- Be able and willing to comply with all study procedures, including magnetic resonance imaging scanning, as per the protocol.
- Must agree to use contraception from baseline until 6 months after the last dose of cladribine tablets, unless they or their partners are infertile or surgically sterile.
- Participants must be aware of all precautions listed in the PI for Mavenclad, and any subsequent disease-modifying therapy treatment received within this clinical study must be adhered to.

**Exclusion criteria**

- Participants must not have a concurrent diagnosis of neurological, psychiatric, or other diseases that, in the opinion of the investigator, could impair the capacity to provide informed consent, interfere with study assessments, or impair the participant’s ability to comply with the study protocol.
- Any contraindication to magnetic resonance imaging scanning.
- Participants who have any contraindications listed on the Australian PI or who have any of the listed precautions listed on the Australian PI.
- The subject is considered by the investigator, for any reason, to be an unsuitable candidate for the trial.

### Assessments

#### NEDA Status

NEDA-3 and NEDA-4 status (absence of clinical relapses, confirmed disability worsening, and new T2 lesions, including brain atrophy [NEDA-4 only]) will be assessed based on the clinical data uploaded into the database (MSBase) [44]. For this study, we will use the NEDA-4 criteria as defined by Kappos [16]. Physical examinations, vital sign assessments, and expanded disability status scale (EDSS) assessments will be performed throughout the study. Concomitant medications and adverse events are assessed at every study visit. On the basis of clinical and MRI parameters, patients with MS will be classified as NEDA-positive (responders) or NEDA-negative (nonresponders).

#### MRI Analysis

3D volumetric sequences will be performed annually in routine clinical practice and before treatment switch. The sequence will be performed on the same 3 tesla MRI scanner with a consistent protocol and without gadolinium administration. The images will be transmitted to the Sydney Neuroimaging Analysis Centre for volumetric analysis.

#### BICAMS Test

Cognition will be measured using BICAMS [30]. This test consists of the oral Symbol Digit Modalities Test (SDMT), the immediate word recall trials of the California Verbal Learning Test-2, and the Brief Visuospatial Memory Test Revised [30]. We chose the BICAMS as a cognitive tool over other available cognitive tests because of its ease of administration over multiple sites and a relatively short time frame in which it can be administered.

#### Serum NfL Chain

Serum NfL (sNfL) will be assessed using the Quanterix Simoa platform. This platform has a specialized immunoassay (NF-light) that allows for the detection of neurological biomarkers at very low levels (pg/mL) with excellent consistency and reproducibility. Given the low levels of sNfL, this was the only assay that could be used for this study at the time of study design.

#### Lymphocyte Subsets

##### Overview

Lymphocyte subset analysis is a composite outcome that combines the results from 2 separate subset panels. Owing to the real-world nature of this study, the surface marker selection was based on the markers available at the local pathology services. The results are shown in Table 1.

**Table 1:** Minimum data set from all sites.

Surface marker			Absolute numbers (IU^a^)		Proportional values		How proportional data is derived
**TBNK^b^ panel**							
	CD45	✓^c^		✓		As per the FBE^d^	
	CD3	✓		✓		CD45+CD3+ (T cells as a percentage of total lymphocytes)	
	CD4	✓		✓		CD45+CD3+CD4+ (CD4+ [T helper class] as a percentage of T cells)	
	CD8	✓		✓		CD45+CD3+CD8+ (CD8+ [cytotoxic T cells] as a percentage of T cells)	
	CD19	✓		✓		CD45+CD3−CD19+ (B cells as a percentage of total lymphocytes)	
	CD56/16	✓		✓		CD45+CD56+CD16+CD3− (natural killer cells as a percentage of total lymphocytes)	
**B memory panel**							
	CD45	✓		✓		As per the FBE	
	CD19	✓		✓		CD45+CD19+ (B cells as a percentage of total lymphocytes)	
	CD27			✓		CD45+CD19+CD27+ (memory B cells as a percentage of total B cells)	
	Immunoglobulin D			✓		CD45+CD19+CD27+IgD±(class switched or unswitched as a percentage of memory B cells)	
	CD38			✓		CD45+CD19+CD27+CD38+ (proportion of plasmablasts as a percentage of memory B cells)	

^a^IU: International Units.

^b^TBNK: T cells, B cells, and natural killer cells.

^c^Tick marks indicate where the subset will be presented as absolute numbers (column 2) or as proportional values (column 3).

^d^FBE: full blood examination.

##### T Cells, B Cells, and Natural Killer Cells Panel

T cells, B cells, and natural killer cells (TBNK) panel will be evaluated at each site using the standardized TBNK cocktail from Becton Dickinson. All sites will use the same antibody cocktail for this panel. All results are presented as absolute numbers and percentages of total lymphocytes. Raw flow cytometry data will be obtained from all sites, and concordance measures will be performed to ensure that the reporting of total cells and subsets of cells is consistent between the sites.

##### B Memory Panel

The second panel will evaluate the B memory cell compartment using antibodies raised against CD45, CD19, CD27, immunoglobulin D, immunoglobulin M, CD21, CD24, and CD38 surface antigens. The minimum panel is gated on the lymphocyte population and assesses CD19, CD27, immunoglobulin D, and CD38. Table 1 lists the minimal data set for each site.

#### DNA Methylation

Epigenome-wide DNA methylation will be evaluated using DNA extracted from frozen whole blood samples. Genomic DNA will be bisulfite converted and hybridized to Illumina Infinium MethylationEPIC BeadChip arrays at the lead site. Changes in DNA methylation (differentially methylated positions) will be expressed as population medians. Medians will be used to identify changes between responders and nonresponders that meet both the significance cutoff of *P*<.05, and a threshold of >10% change.

#### RNA or Gene Expression Analysis

RNA will be collected in PaxGene tubes and stored for future analysis, as per funding.

#### End Points and Outcomes

Participants will attend visits or receive phone calls for assessments at baseline (before 1st dose) and months 1 (before 2nd dose), 3, 7, 12 (before 3rd dose), 13 (before 4th dose), 18, 24, 30, 36, 42, 48, 54, 60, 66, and 72 months, and a variable time point (Tv; at exacerbation of disease and/or before change in treatment or redosing). The schedule of the assessments is shown in Multimedia Appendix 1. BICAMS will be performed annually. MRI will be performed yearly, plus one additional scan 6 months after the first treatment course. Lymphocyte subsets (TBNK and B memory panels) will be performed at screening and at 3, 7, 12, 18, 24, 36, 48, 60, 72, and Tv. Serum NfL will be assessed at screening and at 3, 7, 12, 18, 24, 36, 72, and Tv. DNA methylation and RNA collection occur at screening and at 7, 24, 72, and Tv.

### End Points

The primary end point for all outcome measures is the proportion of patients with MS achieving NEDA status (responders) at 6-years relative to screening. However, preliminary end points will also be considered at each of the major biomarker collection points (months 7, 24, 48, and Tv).

The key outcome measures will be NEDA 3 or 4 responders after 6-years. Additional outcome measures will change over time from baseline in MRI parameters (brain volume loss, lesion load, and lesion volume), cognition, lymphocyte subsets, sNfL, and global DNA methylation profiles. In addition, the Tv time point will be used to determine if there are any changes in biomarker status that may predict disease activity. This will be compared with the time point at which the patient is deemed to have stable disease.

### Statistical Methods

#### Sample Size Calculation

This is a longitudinal study of 150 patients with MS entering the study and predicted to have no more than 20% dropout. Over the study period, we expected 120 patients with MS with longitudinal data for all measured factors: clinical, cellular, and omics. On the basis of CLARITY and CLARITY extension study data, we expect approximately 50% of patients with MS (n=60) to exhibit disease activity according to NEDA 3 (ie, nonresponders) [7-10].

We performed a detailed simulation analysis to assess the power of our sample sizes to detect significant associations at a range of effect sizes and for a range of significance thresholds as per the recommendations of Tsai and Bell [45]. With 120 cases (60 responders vs 60 nonresponders), this study will have 80% power to detect a minimum mean biomarker difference of 10% at a Benjamini-Hochberg false discovery rate of 0.05, which will reasonably balance type I and type II errors. To detect more minor differences, we will use a penalized regression analysis within a machine learning framework using the GLMNet tool [46]. This method is designed for studies with large numbers of predictors and will further enhance the power to identify multi-marker signatures that are predictive of patient response (see details below in the *Association Modeling* section).

#### Missing Data

The trial data set comprises multiple outcome assessments made for each subject over a 72-month period. Therefore, because of the longitudinal nature of the data and the lengthy follow-up period, it is likely that missing outcome data will be present because of loss to follow-up. Patterns and degrees of missingness will be summarized and will inform the approach taken to deal with missing data (eg, missing at random analysis).

#### Association Modeling

Our primary outcome is treatment response status according to NEDA 3. We will perform parametric statistical analysis to determine whether any clinical parameters are associated with outcome—the dependent variable. Multiple repeated measurements of the same individual will be obtained. Therefore, we will apply generalized linear mixed models for binary outcomes, with random effects for time points to account for within-subject correlations and clustering. Clinical factors included in the primary analysis will be EDSS, MRI, and relapse rate measures regressed at baseline. Confounding factors (age, sex, disease duration, etc) will be included as required. By carefully defining covariate values in terms of lagged or leading indicators of study factors, longitudinal data will help us to establish the direction of causation and any lags involved. We will also use Mendelian randomization techniques to help dissect causation from the correlation of evidence for causality. In this analysis, all predictor biomarker variables will be modeled individually and yield a test-specific (unadjusted) *P* value. However, we will apply a Benjamini-Hochberg false discovery rate to adjust the raw *P* value for multiple testing while maintaining power. The secondary outcome variables will be modeled similarly using mixed models with specific parameters dependent on the type of data.

To identify multi-biomarker signatures that predict response in this patient cohort, we will also use machine learning algorithms on the patient cohort data set. Specifically, elastic-net regularized generalized linear model analyses using logistic, linear, or multinomial or Poisson models (depending on the outcome variable) will be conducted within a cross-validation routine to avoid overfitting. This will be performed on the entire factor set using the GLMNet package in the R program [46]. Briefly, GLMNet fits a generalized linear model via penalized maximum likelihood and is akin to a stepwise forward regression with the added feature of being able to perform internal cross-validation. GLMNet allows for both binary and multinomial outcomes. GLMNet allows for the rapid discovery of reduced factor panels that are likely to be associated with outcomes. This complementary approach is not susceptible to multiple testing burdens and will facilitate the identification of the best fitting multifactor signature that is predictive of treatment response [46].

The first analysis time point will be the 6-month assessment, which will be analyzed at this stage. The full data set up to and including the 6-month assessment will be subject to appropriate data cleaning for all variables involved in the primary analysis, consisting of postentry validation checks, and assessing the data for outliers.

Additional interim analysis will be completed at 24 months, 48 months, and again at study termination when all data have been collected.

## Results

### Funding

This study was funded in October 2018 as an investigator-initiated trial by Merck Healthcare Pty Ltd, KGgA, Darmstadt, Germany, to the lead site (John Hunter Hospital).

### Ethics Statement

This study is registered with the Australian and New Zealand Clinical Trials Registry (ACTRN12619000257167) with Universal Trial Number (U1111-1228-2165). Ethical approval for conducting this study was granted on November 8, 2018, by the Hunter New England Local Health District Human Research Ethics Committee (protocol 2019/ETH08849). The study will be conducted in accordance with the revised Declaration of Helsinki, and all participants will provide written informed consent to participate in this study.

### Enrollment

As of May 2021, 145 participants have been enrolled in the study. Recruitment was completed by June 2021, and the study is expected to conclude in 2027.

## Discussion

### Principal Findings

Our study is designed to collect real-world outcomes, while filling the gaps of the previous randomized controlled trials (RCTs) of cladribine tablets for the treatment of MS. This study also offers the opportunity to assess the long-term use of cladribine tablets in a clinical setting and the effectiveness of additional courses of cladribine tablets based on disease activity, in ongoing treatment of MS.

RCTs are the *gold standard* for evaluating treatment outcomes and providing efficacy data for new treatments. However, the strict inclusion criteria and protocol-driven approach may lead to low generalizability because these trials are not always reflective of real-life care [47,48]. In addition, the frequency of MRI in MS RCTs is often higher than that in routine clinical practice [47,48]. Real-world studies can complement data from RCTs by investigating patients who are receiving treatment and being monitored according to routine clinical practice [47,48], and the importance of real-world clinical data is becoming increasingly recognized. For example, MSBase, which is now following over 70,000 patients with MS worldwide with regular assessments, has shaped clinical decision-making in the MS field over the past 5 years using real-world data [49].

The exclusion criteria for this study are deliberately minimal, so that most patients with MS who are planning to commence or choose to take cladribine tablets as part of their routine MS clinical care, are eligible to participate. This study imposes no limits on prior DMT exposure or immunosuppression, no upper limit on EDSS, and an upper age limit of 70. These relaxed inclusion criteria will allow us to capture the broadest range of patients with MS possible, and with the generation of data sets that are reflective of *real-world* MS demographics.

In the CLARITY study, 75% of the participants were treatment-naïve before entering [7]. As with all pharmaceutical trials, there were restrictions on prior DMT use (no more than two failed DMTs were allowed before study entry, and anyone who had used prior immunosuppression was excluded) [7]. This leaves a scarcity of data relating to how patients with MS who have been on prior DMTs, particularly immune suppressants with long-term effects such as alemtuzumab or even dimethyl fumarate, will respond to cladribine tablets. In addition, this does not reflect clinical reality, where many patients with MS have trialled several treatments and often failed several other DMTs before starting cladribine tablets. Data on the safety and effectiveness of cladribine after other DMTs are needed for clinicians and patients with MS to be able to make appropriate decisions about therapy, particularly if they are switching from a prior immunosuppressant. Our study is well-positioned to provide such data.

This trial will also investigate the effectiveness of additional courses of cladribine tablets based on disease activity. Alemtuzumab, another immunomodulatory therapy, is administered in a similar dosing scheme to cladribine tablets [12]. In the CARE-MS II trial, up to four additional courses of alemtuzumab were given to patients with MS as required, with success [5]. Starting in year 3, if patients with MS in this trial have a clinical or radiological relapse, they will be offered an additional course of cladribine. Although the CLARITY extension trial found that additional courses provided no additional benefit, these courses were not offered based on disease activity [9]. This will be the first investigation into an additional cladribine dose based on disease activity.

There is evidence that clinicians are shifting their treatment goals away from relapse free to achieving NEDA status. For example, a recent study using MSBase data demonstrated that just one new subclinical T2 lesion was associated with 1.62 times odds of changing treatment compared with no new lesions [50]. Patients with MS taking cladribine tablets achieved 47% NEDA 3 rates in CLARITY after 2 years [7]; however, rates of NEDA 4 and data beyond 2 years have not been reported. The addition of accelerated brain volume loss to the NEDA criteria (NEDA 4) is an important parameter as it has been shown to be predictive of long-term disability progression and cognitive decline [26,51]. This is highlighted in the FREEDOM trials, where 31% of patients with MS on fingolimod sustained NEDA 3 status, but only 19.7% achieved NEDA 4 after 2 years [16].

Achieving NEDA status may also differ outside the bounds of RCTs. A real-world study conducted in the USA (MS-MRIUS) evaluated nearly 600 patients with MS on fingolimod and found that NEDA 3 was achieved in approximately 58.7% of patients with MS, and 37.2% had achieved NEDA 4 [52]. The differences may be because of the shorter follow-up time (16 months vs 24 months), different patient populations (less severe disease course), or may be reflective of the real-world nature of the data collection versus an RCT [52]. Another real-world study also reported slightly higher levels of NEDA 3 (44%) after 2 years, but did not evaluate NEDA 4 [53]. There has been one small study of cladribine tablets from MSBase data, which compared outcomes over 1 year [54]. Although this study was shorter than CLARITY, the data were similar, with effects lasting at least 4 years [54] despite the majority of patients with MS receiving only one course of treatment. The MSBase study did not specifically report on NEDA 3 outcomes; therefore, it will be interesting to see if NEDA rates remain the same for cladribine use in the clinical setting.

There have been recent criticisms of the current NEDA criteria [55-57]. Stangel et al [57] suggested an algorithm that incorporates cognitive (SDMT), patient-reported outcomes, depression and anxiety ratings, and other parameters. Other criticisms suggest that biomarkers of inflammation and neurodegeneration in body fluids need to be added for a true reflection of disease activity (NEDA-5 or minimal evidence of disease activity) [56]. The inclusion of blood-based biomarkers in this study, including sNfL, as well as cognitive assessments (the BICAMS includes the SDMT) will ensure that we are also able to assess disease activity based on these parameters and assess NEDA5 or minimal evidence of disease activity when a consensus is reached. Furthermore, the discovery of biomarkers that may indicate a response to treatment may allow us to prevent patients with MS from undergoing life-long immune suppression if not required. This could be translated to other MS therapies.

There are minimal real-world data on the effectiveness of cladribine tablets for MS. This study will not only fill this knowledge gap, but also evaluate the efficacy of a clinically indicated additional course beyond the year 2 course. The regular collection of biospecimens for biomarker assessment will help identify biomarkers that may be indicators of treatment response and the need for additional dosing. This may help move clinical practices toward individualized dosing schedules.

### Data Sharing Statement

Deidentified individual data sets will be available upon request to the authors following the publication of the results of the study.

## Appendix

Multimedia Appendix 1 Study overview. Schedule of visits for study duration. The shaded area indicates when each study assessment was performed. Tv=clinical deterioration, Tv+=one month post redose (if applicable), Time point T15* month number is dependent on the year 2 dose (3 months post year 2 week 1). The Brief Cognitive Assessment for Multiple Sclerosis assessment will alternate between the standard and alternate versions. **Indicates magnetic resonance imaging scans to be used only if clinically applicable. X denotes the major analysis time point.

## Abbreviations

BICAMS: Brief Cognitive Assessment for Multiple Sclerosis
CARE-MS Study: Comparison of Alemtuzumab and Rebif Efficacy in Multiple Sclerosis Study
DMT: disease-modifying therapy
EDSS: expanded disability status scale
MRI: magnetic resonance imaging
MS: multiple sclerosis
NEDA: no evidence of disease activity
NfL: neurofilament
RCT: randomized controlled trial
SDMT: Symbol Digit Modalities Test
TBNK: T cells, B cells, and natural killer cells
Tv: variable time point
