# Supplementary figures and images for: Efficacy of Cladribine Tablets as a Treatment for People With Multiple Sclerosis: Protocol for the CLOBAS Study (Cladribine, a Multicenter, Long-term Efficacy and Biomarker Australian Study)
Source: JMIR Res Protoc. 2021 Oct 19;10(10):e24969. doi: 10.2196/24969 (PMC8564661; doi:10.2196/24969)

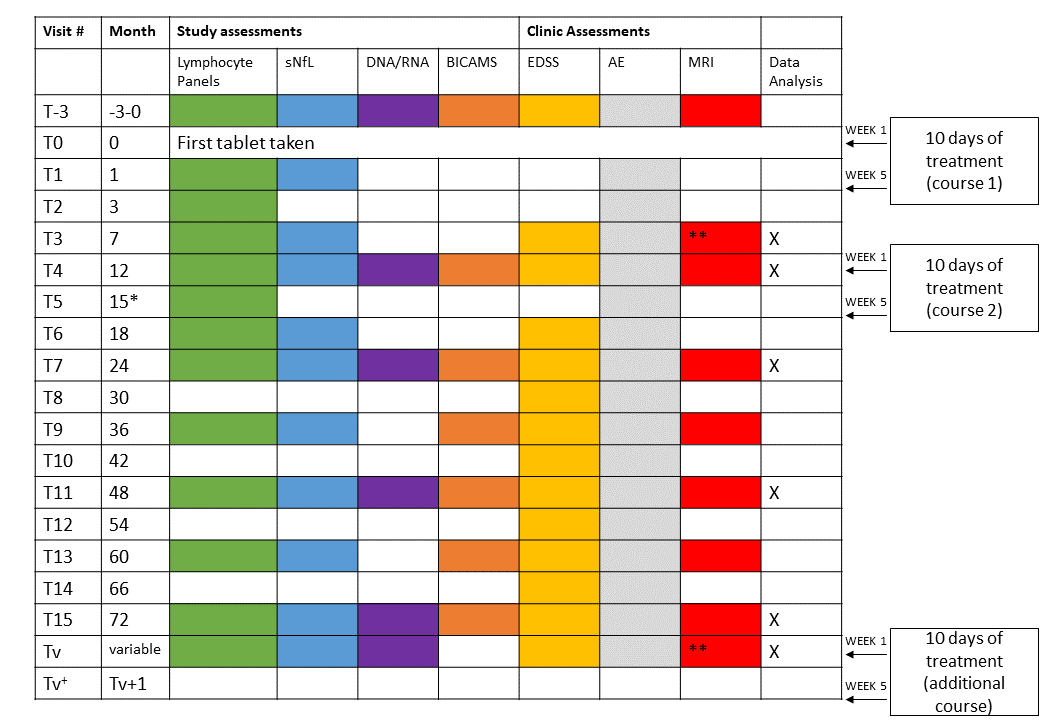

Supplement: Multimedia Appendix 1 [file resprot_v10i10e24969_app1.png]
